# The economics of managing evolution

**DOI:** 10.1371/journal.pbio.3001409

**Published:** 2021-11-16

**Authors:** Troy Day, David A. Kennedy, Andrew F. Read, David McAdams

**Affiliations:** 1 Department of Mathematics and Statistics, Queen’s University, Kingston, Canada; 2 Center for Infectious Disease Dynamics, Department of Biology, The Pennsylvania State University, State College, Pennsylvania, United States of America; 3 Department of Entomology, The Pennsylvania State University, State College, Pennsylvania, United States of America; 4 Fuqua School of Business, Duke University, Durham, North Carolina, United States of America; 5 Department of Economics, Duke University, Durham, North Carolina, United States of America; University of Cambridge, UNITED KINGDOM

## Abstract

Humans are altering biological systems at unprecedented rates, and these alterations often have longer-term evolutionary impacts. Most obvious is the spread of resistance to pesticides and antibiotics. There are a wide variety of management strategies available to slow this evolution, and there are many reasons for using them. In this paper, we focus on the economic aspects of evolution management and ask: When is it economically beneficial for an individual decision-maker to invest in evolution management? We derive a simple dimensionless inequality showing that it is cost-effective to manage evolution when the percentage increase in the effective life span of the biological resource that management generates is larger than the percentage increase in annual profit that could be obtained by not managing evolution. We show how this inequality can be used to determine optimal investment choices for single decision-makers, to determine Nash equilibrium investment choices for multiple interacting decision-makers, and to examine how these equilibrium choices respond to regulatory interventions aimed at stimulating investment in evolution management. Our results are illustrated with examples involving *Bacillus thuringiensis* (Bt) crops and antibiotic use in fish farming.

## Abstract 2

Humans are altering biological systems at unprecedented rates and these alterations often have longer-term evolutionary impacts, such as the spread of resistance to pesticides and antibiotics. In this study, a simple mathematical criterion is derived determining when it is economically beneficial to invest in strategies for controlling and managing evolutionary change.

## 1. Introduction

Evolutionary change in response to human actions is an increasingly important problem [1]. The issues are particularly acute in the context of biological resource management. For example, in aquaculture and agriculture, farmers are sometimes faced with a decision of whether to forgo some amount of current profit in order to implement practices that reduce the risk of problematic evolution. A well-known example is the implementation of refuges for genetically modified crops [2–5]. Plants engineered to produce *Bacillus thuringiensis* (Bt) toxins can be overwhelmed by the evolution of insects resistant to Bt in a decade or less. However, if sufficiently abundant non-Bt crops are planted as well, so that Bt susceptible insects have a refuge, then the evolution of Bt resistance can be delayed for 2 decades or more [6,7]. The downside of this approach is that refuges entail immediate costs because non-Bt crops are not protected against insect pests. Similar issues arise when the harvesting of a biological population drives evolutionary change that reduces its future potential value (e.g., [8–10]). Other examples in a healthcare context include the short-term costs of adding a partner drug to a formulation to increase the useful life span of a novel antimicrobial (e.g., artemisinin combination therapy in malaria [11]) and the implementation of antibiotic stewardship policies in hospitals, which are designed to slow the spread of resistance but entail up-front costs (e.g., hiring additional pharmacists to implement more stringent stewardship policies) [12].

**Fig 1:**
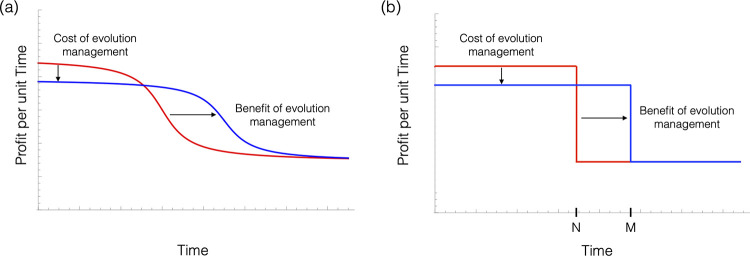
Schematic diagram of evolution management. Plots show profit per unit time in the absence (red) and presence (blue) of evolution management. Both curves decrease over time because adverse evolution reduces profitability. **(a)** Management (blue) entails a reduction of current profit per unit time but has the benefit of prolonging the time until evolution occurs, as compared with no management (red). **(b)** A model based on the idea of a regime shift that approximates panel (a) by treating evolutionary change as a single jump (at time “N” without evolution management and at time “M” with evolution management).

These examples all share a common theme. There is a suite of actions from which a manager must choose, and the economically optimal choice in the short term differs from the choice that minimizes the likelihood of causing adverse evolution. Since such evolution has negative future economic repercussions, we need to know how to take the potential for evolution into account when making economic decisions. Put plainly, when will it be in a manager’s economic interest to invest in evolution management or “stewardship”? Such stewardship reduces profitability in the short term but, by delaying evolution, allows profits to be maintained for a longer period of time (Fig 1).

The role of biological evolution in economic decision-making has received considerable attention [13,14], with previous studies examining a wide range of important topics from antibiotic resistance (e.g., [15–19]) to the role of harvesting on the evolution of fish populations (e.g., [20,21]), to the role of refuge sizes in Bt crops for preventing resistance in pest species [2,3,5,22–25]. Most of these studies employ detailed and system-specific mathematical models, making it difficult to appreciate the underlying commonalities shared by all problems involving evolution management. In this paper, we therefore develop a generic model. To do so, we borrow ideas from the economic literature on regime shift and catastrophes [26–35] because this framework provides a general yet straightforward way to model the economic consequences of evolutionary change (Fig 1B). The end result is a simple dimensionless inequality showing that managing evolution is beneficial if the percentage increase in the effective life span of the biological resource that management provides is larger than percentage increase in annual profit that could be obtained by not managing evolution. We show how this inequality can be used to determine optimal investment choices for single decision-makers, to determine Nash equilibrium investment choices for multiple interacting decision-makers, and to examine how these equilibrium choices respond to regulatory interventions aimed at stimulating investment in evolution management.

## 2. A model of evolution management

Suppose that time is measured in discrete bouts such as years and that a manager must choose some action *γ* during each year. For example, a farmer might need to decide what fraction (0≤*γ*≤1) of a farm to plant with non-Bt crops, or a physician might need to decide whether (*γ* = 1) or not (*γ* = 0) to include a partner drug with a novel antimicrobial. The manager’s goal is to choose *γ* to maximize profit, where “profit” captures whatever is the manager’s objective. For a hospital adminstrator making decisions that affect antibiotic-prescribing practices, for instance, “profit” would naturally include patient welfare, in addition to the financial impacts of those practices on the hospital itself.

The trade-off between present and future gains is at the heart of our analysis. To capture this trade-off, we express all profits in terms of “present value” [36]. For example, if *r* is the interest rate (compounded annually), then *F* dollars invested today will grow to *F*(1+*r*)^*t*^ dollars *t* years from now. Equivalently, *F* dollars earned *t* years from now has a present value of *F*/(1+*r*)^*t*^. If we let *δ*≡1/(1+*r*) denote the annual factor by which future profits are discounted, then an indefinite profit stream of *F* dollars per year has a total present value of *F*+*Fδ*+*Fδ*^2^+⋯ = *F*/(1−*δ*). The factor *L*≡1/(1−*δ*) can be thought of as the “effective life span” of an indefinite profit stream. Specifically, *L* is the factor by which the profit from a single year must be multiplied to produce the profit stream’s total present value.

The profitability of a resource such as a farm depends on the evolutionary state of a biological population, for instance, whether the pests infesting a cornfield are Bt resistant or whether the microbial organisms infecting a stock of fish are antibiotic resistant, etc. We model evolution in a simple way, borrowing from the economic literature on regime shift and/or “catastrophes” such as climate change, depletion of groundwater resources, fisheries collapses, and the accumulation of pollution [26–35,37–40]. We suppose that the population is in one of two possible states: (1) adverse evolution has not yet occurred, referred to as the “pristine” state; and (2) adverse evolution has occurred, referred to as the “evolved” state. We assume that the process of evolution is irreversible (Fig 1B). Supporting information A in S1 File illustrates how one can relax the assumption of irreversibility.

We use Π(*γ*) and Π to denote the profit obtained per year while in the pristine and evolved states, respectively, where the former depends on the evolution management action *γ* chosen while in the pristine state. Once in the evolved state, we assume that the manager no longer employs a stewardship strategy since the process of evolution is irreversible. We assume that the maximum possible profit while in the pristine state is larger than that while in the evolved state, i.e., $\underset{\gamma }{\max}\;\Pi (\gamma )>\underline{\Pi }$, implying that evolutionary change is adverse from an economic standpoint. We define *E*(*γ*)≡Π(*γ*)−Π to be the excess profit per year that comes from being in the pristine state when the manager chooses action *γ*. Thus, there is a guaranteed baseline profit stream of Π per year regardless of the state of the system, as well as an excess profit stream of *E*(*γ*) per year that depends on *γ* and that can be obtained for as long as the system remains in the pristine state. Once adverse evolution occurs, this excess profit stream ends (Fig 1B).

Now, if *κ*(*γ*) represents the annual probability that adverse evolution does not occur (which will depend on the management choice *γ*), then the total present value of the excess profit stream is

$$E(\gamma )+\delta \kappa (\gamma )E(\gamma )+(\delta \kappa (\gamma ){)}^{2}E(\gamma )+\cdots =E(\gamma )L(\gamma ),$$ (1)

where *L*(*γ*) = 1/(1−*δκ*(*γ*)) can be viewed as the effective life span of this excess profit stream. Given action *γ*, an excess profit of *E*(*γ*) is obtained each year (over and above the baseline Π) for an effective number of *L*(*γ*) years. The manager should choose whatever action *γ* maximizes (1) (Supporting information A in S1 File).

Note that the effective life span *L*(*γ*) incorporates two components of discounting: economic discounting, *δ*, due to the time value of money, and evolutionary discounting, *κ*(*γ*), due to the possibility that excess profits will end because of adverse evolution. Evolutionary discounting is an example of what economists refer to as “endogenous discounting” [27,32,35,39], whereby the rate of discounting depends on the choices of economic agents.

## 3. Results

**Fig 2:**
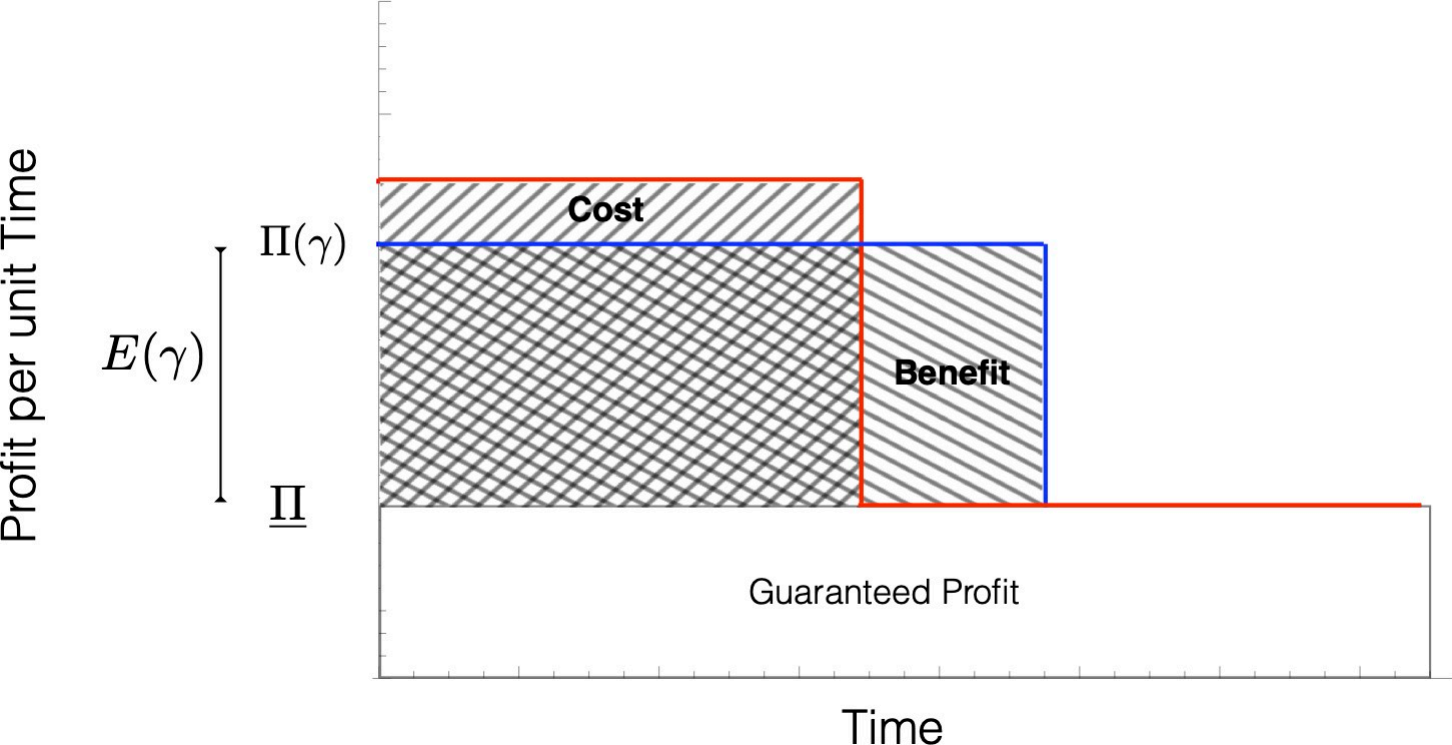
Evolution management when evolution is modeled as a regime shift. Plots show profit per unit time (“annual profit”) with no stewardship (red) and with stewardship (blue). Π(*γ*) is annual profit while in the pristine state with management strategy *γ*, Π is annual profit while in the evolved state, and the excess annual profit is *E*(*γ*) = Π(*γ*)−Π. Guaranteed profit is the profit obtainable no matter which strategy is followed. Hatched areas are a schematic of total excess profit achievable from an evolution management strategy (blue) or not (red). Nonoverlapping areas represent the cost and benefit (in terms of excess profit) of evolution management. Note that hatched areas are only schematic because they do not reflect how all excess profits must be expressed in terms of present value. Hatched areas are exact representations of the cost and benefit in the case where there is no discounting.

We can see from expression (1) that, in the absence of evolution (i.e., if *κ* = 1), it is optimal to choose *γ* to maximize *E*(*γ*). We refer to this choice of *γ* as the “no stewardship” choice and denote it by *γ*_0_, meaning zero investment in evolution management. If evolution can occur, however, then the manager faces a trade-off between increasing excess profits *E*(*γ*) today and extending the expected length of time *L*(*γ*) spent in the pristine state (Fig 2).

How much should one be willing to sacrifice, in terms of excess profit per year, in order to manage evolution? Any management action *γ* different from *γ*_0_ will be beneficial if *E*(*γ*)*L*(*γ*)>*E*(*γ*_0_)*L*(*γ*_0_). This inequality can be rearranged into the dimensionless form

$$\frac{L(\gamma )-L(\gamma_{0})}{L(\gamma_{0})}>\frac{E(\gamma_{0})-E(\gamma )}{E(\gamma )}$$ (2)

which we refer to as “the evolution management inequality.” The left-hand side of (2) is the percentage increase in the effective life span of the excess profit stream that comes from managing evolution. The right-hand side of (2) is the percentage increase in annual excess profit that comes from not managing evolution. Inequality (2) says that an evolution management action *γ* will be better than the no stewardship choice *γ*_0_ if the percentage increase in the effective life span of the biological resource from choosing *γ*, due to delaying evolution, is greater than the annual percentage increase in excess profit that could be obtained by using *γ*_0_ instead. We can thus think of the left-hand side of inequality (2) as the percentage benefit of evolution management, *B*, and right-hand side as the percentage cost of evolution management, *C* (Fig 2).

### 3.1 Isolated managers

Suppose there is a single manager or, equivalently, that there are multiple managers whose choices have no impact on one another. The manager finds stewardship action *γ* more profitable than no stewardship, *γ*_0_, so long as the evolution management inequality (2) holds. Because $L(\gamma )=\frac{1}{1-\delta \kappa (\gamma )}$ and $E(\gamma )=\Pi (\gamma )-\bar{\Pi }$, we can rewrite (2) as

$$\delta \frac{\kappa (\gamma )-\kappa (\gamma_{0})}{1-\delta \kappa (\gamma )}>\frac{\Pi (\gamma_{0})-\Pi (\gamma )}{\Pi (\gamma )-\underline{\Pi }}$$ (3)

Three insights about stewardship investment incentives emerge from inequality (3):

1. **Lower discounting increases the incentive for stewardship.** If discounting is complete, meaning that there is no value placed on future profit (i.e., *δ* = 0), then costly evolution management is never economically advantageous because the left-hand side of (3) is 0. As *δ* increases, meaning that there is less discounting of future profit, the left-hand side of (3) increases. The percentage change in the effective life span that management strategy *γ* provides increases because future profit is more valued. As a result, one should be willing to pay a larger amount for evolution management. This also means that, for any fixed cost of evolution management (i.e., any fixed value of the right-hand side of (3)), there is a critical discount factor δ* above which stewardship action *γ* is better than no stewardship, *γ*_0_.
2. **More effective management increases the incentive for stewardship.** If one’s actions do not affect evolution (i.e., if *κ*(*γ*) = *κ*(*γ*_0_)), then evolution management is clearly never advantageous (again the left-hand side of (3) is 0). As the effect of one’s actions on evolution increases (i.e., as *κ*(*γ*)−*κ*(*γ*_0_) increases for any fixed *γ*), one should be willing to pay an increasing amount for evolution management.
3. **Lower profit in the evolved state increases the incentive for stewardship.** As the annual profit in the evolved state Π decreases, the percentage cost of evolution management (the right-hand side of (3)) decreases. This means that the incentive for stewardship increases. This is intuitively reasonable since, if very little profit can be obtained once evolution has occurred, then the loss in profit that comes from managing evolution will pale in comparison to the loss in profit that comes from the evolutionary degradation of the biological resource. Thus, for example, the costs of future drugs, insecticides, and crop varieties should factor in when deciding how to use a particular product now. Failing first-generation single-toxin Bt crops are being replaced by more expensive crop varieties engineered to produce two or more new toxins (so-called pyramid crops [7]). If these next lines of defense are very expensive, then management today will be more attractive.

#### Example: Bt corn refuges

Insect pests like the European corn borer (*Ostrinia nubilalis*) and other lepidopteran species can cause significant financial losses in corn farming [4,5,25]. Bt corn is a genetically modified plant that expresses toxic proteins from the bacterium *Bacillus thuringiensis* as a means of controlling these insects. However, insects can evolve resistance to the toxic proteins [6]. This evolution can be slowed (and so the useful life span of the engineered crop prolonged) if farmers who wish to plant Bt corn set aside a certain fraction of their land for non-Bt crops (called a refuge). The rationale is that non-Bt crops will support pests that are sensitive to Bt, and mating between these insects and those from the Bt crops will hinder the spread of resistance [7].

Non-Bt crops are often less profitable than Bt crops because non-Bt crops are susceptible to loss of yield through pest infestation. A farmer’s short-term financial interests are thus often best served by planting only Bt crops. However, setting aside a refuge for non-Bt crops is an evolution management strategy that might slow the spread of resistance and so increase the effective life span of a farmer’s resistance-free profit stream. Thus, although farmers can be incentivized through taxation or by mandate to implement evolution management practices [41], it might actually be in their economic interest to do so without such incentives.

The evolution management inequality can be used to gain a better understanding of when this will be the case. In accordance with the general model, we treat the spread of Bt resistance as a “regime shift” and assume that, once Bt resistance has spread, only non-Bt crops are a viable farming option. For example, 3 years after the commercial use of a Bt-engineered corn variety began in Puerto Rico, resistance evolution in fall army worms (*Spodoptera frugiperda*) had reduced the efficacy of the engineered corn to such an extent that the manufacturer voluntarily withdrew it from the market. High levels of Bt resistance persisted for at least 4 years after product withdrawal [6].

Whether a refuge is economically worthwhile to the farmer is determined by inequality (2), where we interpret *γ* to be the fraction of land set aside as a refuge. In the absence of evolution, the optimal choice is to plant only Bt corn (i.e., *γ*_0_ = 0). Furthermore, assuming that the farmer will plant non-Bt corn after Bt resistance has spread, the right-hand side of (2) reduces to the simple expression *γ*/(1−*γ*) (Supporting information B in S1 File, Section 1). Specifically, *γ*/(1−*γ*) is the percentage increase in annual excess profit that could be obtained by taking the refuge of size *γ* and planting Bt corn there instead. Planting a non-Bt crop in this refuge will therefore be advantageous, provided that the percentage increase in effective life span of the resistance-free excess profit stream that comes from doing so is larger than *γ*/(1−*γ*). For example, a 20% refuge will be advantageous only if it increases the effective life span of the resistance-free excess profit stream by at least 0.2/(1–0.2) or 25%. For instance, if the effective life span of the resistance-free excess profit stream is 8 years without a refuge, then a 20% refuge will be economically beneficial provided it increases the effective life span by at least 0.25×8 = 2 years. Such increases appear to be biologically attainable [7,42].

More generally, if the effective life span of the resistance-free excess profit stream is *L* years without a refuge, then setting aside a fraction *γ* of the land for non-Bt crops will be economically beneficial provided that it increases the resistance-free effective life span of the farm by at least *Lγ*/(1−*γ*) years. This simple result depends on the assumption that the farmer will continue to plant (non-Bt) corn after Bt resistance has spread but, remarkably, is otherwise independent of the specific costs or revenues per acre. This provides an explanation for Hurley and colleagues’ [2,3] finding that the optimal refuge size is relatively insensitive to costs and revenues since, in our analysis, the costs and revenues completely drop out of the equation.

If instead the farmer has more profitable options for land use after resistance has emerged than continuing to farm corn, then the incentive to invest in stewardship will be reduced, and it might even be optimal to plant no refuge at all during the pristine phase (Supporting information B in S1 File, Section 1). Thus, whether a farmer finds it beneficial to engage in stewardship depends critically on the profitability of alternative uses of the farm. Also note that the above analysis considers the case of a binary choice between a certain level of stewardship (defined by a fixed refuge size) or none at all and determines how effective evolution management must be for the refuge to be economically beneficial. We could, alternatively, use inequality (2) to determine the optimal refuge size from among a continuum of choices. Supporting information C in S1 File illustrates how this can be done in the context of a different example involving fish farming. We also note that the effectiveness of management, *κ*, will often depend on the management choices of other actors as well. We turn to analyzing such cases next.

### 3.2 Interacting managers

The stewardship decisions of one manager often affect the incentives driving other managers’ stewardship decisions. For example, if one hospital takes steps to reduce the evolutionary emergence of antibiotic resistance in their hospital, then this might also reduce the prevalence of resistance in the broader community, indirectly altering other hospitals’ incentives to engage in stewardship.

To account for such interactions, we can again use inequality (2), but now we must allow both *E* and *L* to depend on the actions chosen by other managers. Consider the case where each manager is choosing between a fixed stewardship level, *γ*, and no stewardship, *γ*_0_. Furthermore, assume that both *E* and *L* depend on the fraction, *p*, of other managers who engage in stewardship. To make this clear, we introduce a subscript *p* on the functions *E* and *L* (the functions *E* and *L* will also typically depend on how many of the managed resources are in the pristine state, a nuance considered further in Supporting information D in S1 File).

For ease of notation, let $B(p)\equiv \frac{L_{p}(\gamma )-L_{p}(\gamma_{0})}{L_{p}(\gamma_{0})}$ and $C(p)\equiv \frac{E_{p}(\gamma_{0})-E_{p}(\gamma )}{E_{p}(\gamma )}$ be, respectively, the percentage benefit and percentage cost of stewardship. By inequality (2), an individual manager will find stewardship profitable if

B(p)>C(p). (4)

**Fig 3:**
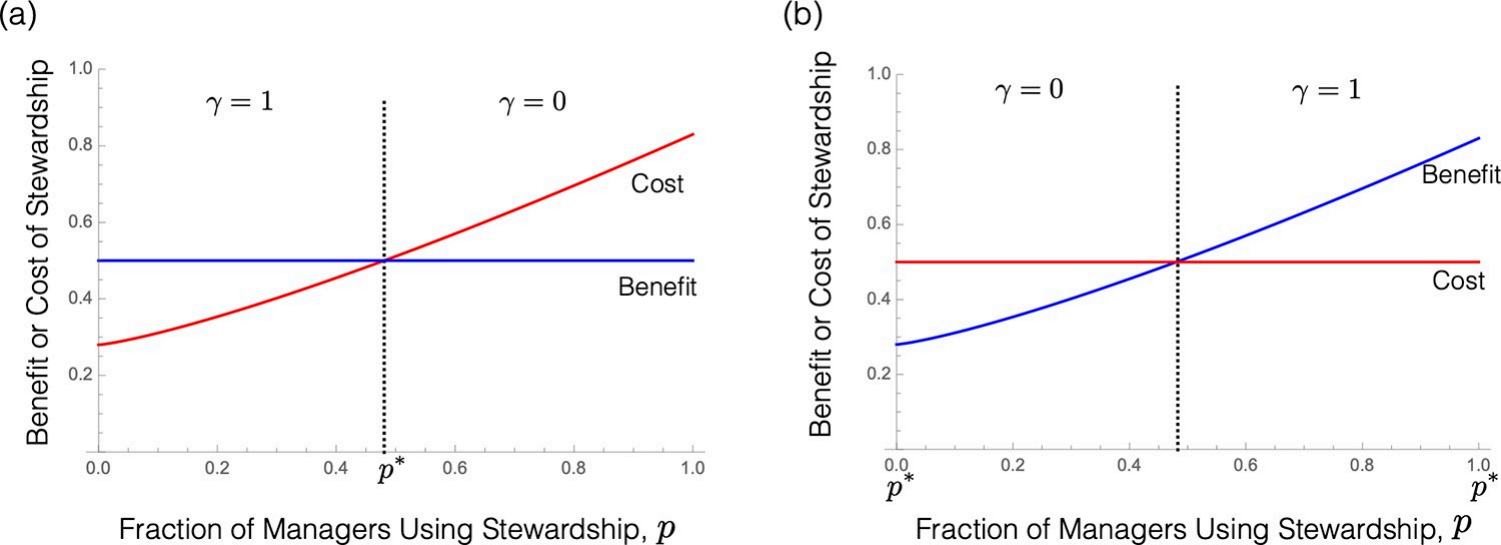
The percentage benefit of stewardship (left-hand side of inequality (4); blue) and the percentage cost of stewardship (right-hand side of inequality (4); red) are plotted as a function of the fraction of managers who are engaging in stewardship, *p*. Nash equilibria that are also convergence stable are indicated by p* (see Supporting information E in S1 File, Section 1). **(a)** An example in which *B*(*p*)−*C*(*p*) is decreasing in *p* (“negative strategic feedback”). There is a unique Nash equilibrium, in which some managers engage in stewardship and others do not. **(b)** An example in which *B*(*p*)−*C*(*p*) is increasing in *p* (“positive strategic feedback”). There are 2 convergence stable Nash equilibria: one in which no managers engage in stewardship and another in which all managers do.

We focus in the main text on two special cases (a complete analysis is in Supporting information E in S1 File):

1. **More investment by others discourages individual investment (“negative strategic feedback”).** When *B*(*p*)−*C*(*p*) decreases as *p* increases, as in Fig 3A, more investment by others decreases each manager’s individual incentive to invest. This can happen, for instance, if the cost of evolution management services increases as more managers engage in evolution management. In this case, anything that causes some managers to engage in stewardship will, in turn, tend to induce other managers not to engage in stewardship, a negative strategic feedback (economists refer to the game among managers in this case as exhibiting “strategic substitutes” [43]). An explicit example is given below.
2. **More investment by others encourages individual investment (“positive strategic feedback”).** When *B*(*p*)−*C*(*p*) increases as *p* increases, as in Fig 3B, more investment by others increases each manager’s individual incentive to invest. This can happen, for instance, when there are synergies between managers’ investments and/or when there are population-wide economies of scale associated with stewardship. In this case, anything that causes some managers to engage in stewardship will increase others’ incentive to do the same, a positive strategic feedback (economists refer to the game among managers in this case as exhibiting “strategic complements” [43]).

#### Example: Bt corn refuges

We illustrate the two abovementioned possibilities by again considering the example of Bt corn. First, consider a situation in which, as the fraction of farmers employing a Bt refuge increases, the overall abundance of nonresistant pest insects increases as well. As a result, the percentage cost of using a refuge for any given farmer (the right-hand side of (4)) might increase as the fraction of farmers employing a refuge increases, because a refuge is then more likely to be infested and thus provide less revenue. If so, each farmer will have less of an incentive to plant a refuge as the fraction of other farmers employing a refuge increases. Fig 3A provides a specific example in which the cost and benefit of stewardship become equal when the fraction of managers investing in stewardship is at an intermediate value p* (see Supporting information B in S1 File, Section 2 for details). In this case, individual managers will find it beneficial to invest in stewardship if few others are doing so, but find it disadvantageous to invest in stewardship if many others are doing so. As a result, collectively, we expect the population of managers as a whole to reach a point where exactly a fraction p* of managers engage in stewardship, fraction (1−p*) do not, and all of them have no incentive to unilaterally alter their action because *B*(p*) = *C*(p*). This is the so-called “Nash equilibrium” of the game being played by farmers, uniquely determined in the negative strategic feedback case by the point where the percentage cost and benefit curves cross (e.g., Fig 3A; Supporting information E in S1 File). At this equilibrium, all managers earn the same overall profit (in present value terms) whether or not they engage in stewardship.

Second, suppose instead that as the fraction of farmers employing a Bt refuge increases, the likelihood of any given farmer importing resistant pests from other farms decreases. As a result, the percentage benefit of using a refuge for any given farmer (the left-hand side of (4)) will increase as the fraction of farmers employing a Bt refuge increases. Fig 3B again provides a specific example in which the cost and benefit of stewardship are equal when the fraction of managers investing in stewardship is at an intermediate value (see Supporting information B in S1 File, Section 2 for details). In this case, however, individual managers find it beneficial to invest in stewardship if many others are already doing so, but find stewardship disadvantageous if few others are doing so. As a result, collectively, we expect the population of managers as a whole to reach one of two Nash equilibria, with either no one engaging in stewardship or everyone engaging in stewardship (e.g., Fig 3B; Supporting information E in S1 File).

### 3.3 Changing the game

In this final section, we consider how an external regulator might alter the strategic landscape to incentivize increased investment in stewardship.

#### Subsidies

Subsidizing stewardship investments is one obvious way to incentivize such investments. Here, we consider two possibilities: paying those who engage in stewardship (i) a small amount each year in perpetuity; or (ii) a large amount for one year only. The qualitative impact of such subsidies depends on whether there is positive or negative strategic feedback among managers’ investments.

Small permanent subsidy. Paying *S*>0 each year to those who engage in stewardship decreases the cost of stewardship and so decreases the right-hand side of inequality (4) for any given *p*. In the negative strategic feedback case, assuming that managers behave according to the Nash equilibrium prior to the subsidy (with fraction p* engaging in stewardship, where *C*(p*) = *B*(p*)), introducing a small subsidy lowers the red cost curve in Fig 3A a small amount. Since now *B*(p*)>*C*(p*), managers who were not previously investing in stewardship will find it in their interest to do so. However, as some managers switch to engage in stewardship, others have less incentive to do so due to negative strategic feedback. Eventually, at the new Nash equilibrium, the proportion of managers who engage in stewardship will be slightly larger than before the subsidy. On the other hand, in the positive feedback case, assuming that managers begin in the no stewardship Nash equilibrium p* = 0, a small subsidy will have no effect at all on managers’ behavior: All will continue not to invest in stewardship because we still have *B*(0)<*C*(0). Intuitively, managers in this case have a strong incentive not to engage in stewardship so long as they believe that others are also not engaging in stewardship. Unless the subsidy is sufficiently large to change their belief about others’ likely behavior, it will have no effect.

Large temporary subsidy. Instead of giving a small permanent subsidy, suppose the regulator were to give a large enough subsidy to make stewardship beneficial for all managers, regardless of the actions of others, but only for a single year. In the year that the subsidy is offered, clearly everyone will be incentivized to engage in stewardship. But what about the following year, when the subsidy is gone? In the negative strategic feedback case (Fig 3A), managers can be expected to return to playing the same (unique) Nash equilibrium that they were before the subsidy. In this case, the temporary subsidy has no long-term incentivizing effect. On the other hand, in the positive strategic feedback case (Fig 3B), the fact that others have now switched their behavior to engage in stewardship could be enough to change managers’ beliefs and thereby change their subsequent behavior. In particular, so long as managers expect that others will continue to engage in stewardship after the subsidy is gone, they will want to do so as well. In this case, the temporary subsidy could have a permanent effect, shifting managers en masse from the no-stewardship equilibrium to the all-stewardship equilibrium.

An interesting implication is that, in this latter case, no subsidy at all may be needed to induce managers to switch from the no-stewardship equilibrium to the all-stewardship equilibrium. So long as the regulator can somehow make managers believe that everyone else will begin engaging in stewardship at some future time, all of them will then have an incentive to start doing so at that time.

#### Evolution management cartels

When managers interact with one another, there can be competing interests at multiple levels, as the interests of an individual manager may differ from the collective interests of all managers (viewed as a group) or of society as a whole. As a result, the highest possible profit for the managers as a whole need not result when they act purely in their own self-interest. Instead, coordinated decision-making among managers might generate even higher profit.

One way coordinated decision-making can be achieved is by allowing managers to form an “evolution management cartel” with the authority to dictate individual managers’ actions. The term “cartel” evokes notions of monopolization or nationalization, but it also includes any sort of collaboration by which individuals in a group can be incentivized or compelled to take actions that benefit the group, such as the myBMP program in Australia [44,45].

If increasing collective stewardship, captured by *p*, increases each individual manager’s profits (i.e., d*E*_*p*_(*γ*)*L*_*p*_(*γ*)/d*p*>0, creating a “positive externality”), then coordinated action among managers will result in each individual manager investing more than they otherwise would have in Nash equilibrium. For example, planting a Bt refuge reduces the amount of corn that a farmer produces, creating a positive externality for other farmers as the price of corn increases when less corn is produced. In that context, farmers capable of collective action would engage in more stewardship, slowing the emergence of Bt resistance while also producing less corn relative to any Nash equilibrium.

## 4. Discussion

Our central finding is the “evolution management inequality,” expressed in three slightly different ways in (Eqs 2–4), which must be satisfied for evolution management to be economically beneficial for the biological resource manager. In words, this inequality says that an evolution management action is individually beneficial if and only if the percentage increase in the effective life span of the biological resource due to evolution management is greater than the annual percentage increase in excess profit that could be obtained by not managing evolution. The former can be viewed as the benefit of evolution management, while the latter can be viewed as the cost (Fig 2).

The simplicity of inequality (2) stands in contrast to previously published studies of specific examples of evolution management. A simple dimensionless condition is possible here because we have treated evolution as an instantaneous and irreversible regime shift. In reality, adverse evolution often occurs slowly over several years, causing both excess profit and evolutionary discounting to change from year to year. When computing the costs and benefits of evolution management, one must therefore integrate these time-varying components using a detailed model that captures how they change. However, such complexity can often be reasonably approximated by assuming a simple regime shift (Fig 1). Using this simplifying assumption, we are able to approximate the total present value of the excess profit stream as the product of a constant annual excess profit and an effective life span, leading to much simpler results.

Increasing the effectiveness of evolution management technology obviously makes stewardship more attractive to managers, but evolution management strategies cannot be judged on their efficacy alone. Strategies that are highly effective at slowing or even completely preventing adverse evolution might be economically unjustifiable for the managers who must implement these strategies. Thus, for instance, it is quite possible to create refuge strategies that preserve the efficacy of a particular Bt crop variety for many decades, but the costs associated with the required refuge may not be economical for farmers. Likewise, resistance management strategies that might substantially extend the useful life span of a novel antibiotic may be of little interest to the companies that develop them. That said, the analyses presented here and in the appendices suggest that evolution management can be economically beneficial under a remarkably wide range of conditions. Previous results [46] have also shown that, if the cost of extending the life span of existing drugs through evolution management is the same as the cost of increasing the rate of new drug development, then investing in management is typically a better option for ensuring the continued availability of effective treatment. The results presented here thus add further strength to the conclusion that investing in evolution management will often be desirable.

Inequality (2) provides useful conceptual insight into the problem of evolution management, but it can also be used to provide quantitative insight. As an example, we considered the case of refuge sizes in Bt crops in detail. We show that inequality (2) delivers several of the key insights obtained from more complex economic models, such as in Hurley and colleagues [2,3]. For example, Hurley and colleagues [2,3] found that total profit was maximized when farms employed a 26% Bt crop refuge, meaning that 26% of crops were non-Bt. This maximized profit in present-day dollars by delaying the evolution of Bt-resistant pests from 15 years to more than 25 years. Inequality (2) shows that this 26% refuge is economically justifiable to individual managers given that it increases the life span of the Bt crop to at least 23 years. Hurley and colleagues [2,3] found, somewhat to their surprise, that the optimal refuge size is relatively insensitive to the costs and revenues of each type of crop. Inequality (2) explains why: In the case of Bt crops, we showed that the optimal refuge size is independent of these costs and revenues. Finally, Hurley and colleagues [2,3] also found that optimal refuge size increases as the planning horizon increases (i.e., as the discounting of future income decreases), similar to our general analysis of inequality (2).

We extended our analysis to settings in which there are multiple managers who interact with one another, each acting in their own self-interest. For example, when a farmer plants a Bt refuge, this might benefit other nearby farmers by preventing the emergence of resistant pests that could then fly to neighboring farms. In this context, managers are playing a “game” in which their individual incentive to invest in stewardship may depend on others’ investment decisions. The evolution management inequality still characterizes when an individual manager will find it beneficial to invest in such games, the difference being that now one must account for how the percentage benefit and percentage cost of stewardship depend on others’ stewardship decisions; see inequality (4).

Our game theoretic analysis focused on two main cases. The first explores a case where investment in evolution management is less attractive when others are also investing in management. Specifically, if the net percentage benefit of stewardship decreases with the fraction of others who engage in stewardship, then managers have less individual incentive to engage in stewardship when more others do so, i.e., there is “negative strategic feedback.” In this case, we showed that there is a unique Nash equilibrium of the evolution management game being played by managers and that regulatory interventions can influence outcomes by changing this Nash equilibrium. The second explores a case where investment in evolution management is more attractive when others are also investing in management. Specifically, if the net percentage benefit of stewardship increases with the fraction of others who engage in stewardship, then managers have more individual incentive to engage in stewardship when more others do so, i.e., there is “positive strategic feedback.” In this case, we showed that the evolution management game can have multiple Nash equilibria and that regulatory interventions can influence outcomes by changing which Nash equilibrium is played. Most strikingly, temporary interventions (such as a large temporary subsidy or a temporary requirement that all managers must engage in stewardship) can have a permanent impact on manager behavior, by shifting managers en masse from an equilibrium in which no one invests to another in which everyone invests.

When assessing the economic burden imposed by evolution management, it is important to distinguish between individual and collective impacts. As emphasized in our game theoretic analysis, managers’ individual and collective incentives can differ, sometimes so much so that a policy that hurts managers individually—in the sense that each manager would rather not abide by the policy—may benefit them all collectively. For example, consider Bt corn farmers’ decisions whether to invest in planting a refuge to delay the emergence of Bt-resistant pests and suppose that planting a refuge benefits other neighboring farmers (creating a “positive externality”) while reducing neighbors’ incentive to plant a refuge themselves (creating a “negative strategic feedback”). Farmers will in Nash equilibrium invest too little, reducing their collective profits while also hastening the rise of Bt resistance. In this context, farmers benefit collectively from interventions that incentivize them to invest more in refuges, e.g., a “revenue neutral subsidy,” whereby farmers are taxed up front and then distributed that money back in the form of a subsidy to those who engage in stewardship. In fact, such a program has been implemented by regulators in Australia, whereby cotton farmers pay an up-front license fee to Monsanto but later receive a rebate for this fee if they have successfully implemented resistance management practices [45]. Such a program should be a “win-win,” benefiting farmers by solving their collective action problem and benefiting Monsanto by extending the life of its patented products.

As another example, consider hospital managers’ decisions whether to invest in containing the spread of drug-resistant *Neisseria gonorrhoeae*. Hospitals in the United States are required to report any suspected cases of drug-resistant *N*. *gonorrhoeae* to the Centers for Disease Control and Prevention (CDC), but can choose whether to incur extra cost to invest in contact tracing efforts to discover additional cases. Let *γ* represent whether (*γ* = 1) or not (*γ* = 0) a hospital invests in contact tracing. Further, suppose that investment by any individual hospital has a negligible impact on the spread of resistance and thus on the length of time, *L*, until resistance becomes common. This means that, in inequality (4), *L*_*p*_(*γ*) depends very little on a manager’s individual decision, *γ*, but is an increasing function of the aggregate level of investment, *p*. The left-hand side of inequality (4) is therefore approximately 0, implying that no hospital manager finds it individually worthwhile to invest in contact tracing. However, hospital managers collectively might benefit by all making such investments at the same time, as doing so increases aggregate investment. There are many ways to achieve such collective action in practice, such as through a resistance fighting joint venture among hospitals in a given region or in response to a government mandate.

We have deliberately focused primarily on the economics of resistance management, but other societal benefits can accrue from evolution management. For instance, the US Environmental Protection Agency originally mandated refuges not to protect the future profit stream for farmers but rather because Bt crops require less chemical insecticide, reducing environmental contamination and protecting organic farms from airborne insecticide drift. Inequality (2) helps define the level of investment which is economically justifiable to those who bear the cost of evolution management, who we have termed “biological resource managers.” To the extent that there are societal benefits that justify investment beyond what these decision-makers find economically justifiable, our analysis can be used to quantify the economic burden imposed on managers by social welfare–maximizing investment.

## Supporting information

S1 File **Supporting information A in S1 File:** Individually optimal stewardship. **Supporting information B in S1 File:** An example involving Bt refuge size. **Supporting information C in S1 File:** An example involving fish farming. **Supporting information D in S1 File:** Manager population dynamics**. Supporting information E in S1 File:** Game theoretic analysis.
(PDF)
