# Supplementary material for: The economics of managing evolution
Source: PLoS Biol. 2021 Nov 16;19(11):e3001409. doi: 10.1371/journal.pbio.3001409 (PMC8594813; doi:10.1371/journal.pbio.3001409)
Supplement: S1 File — Supporting information A in S1 File: Individually optimal stewardship. Supporting information B in S1 File: An example involving Bt refuge size. Supporting information C in S1 File: An example involving fish farming. Supporting information D in S1 File: Manager population dynamics. Supporting information E in S1 File: Game theoretic analysis. (PDF) [file pbio.3001409.s001.pdf]

Supporting Information
The Economics of Managing Evolution

Troy Day[1,*], David A. Kennedy[2], Andrew F. Read[2,3], and David McAdams[4,5]

1. Department of Mathematics and Statistics, Queen's University, Kingston, Canada, day@queensu.ca
2. Center for Infectious Disease Dynamics, Department of Biology, The Pennsylvania State University, University Park, PA, USA
3. Department of Entomology, The Pennsylvania State University, University Park, PA, USA
4. Fuqua School of Business, Duke University, Durham, NC, USA
5. Department of Economics, Duke University, Durham, NC, USA

Contents

A - Individually-optimal stewardship
B - An example involving Bt refuge size
C - An example involving fish farming
D - Manager population dynamics
E - Game-theoretic analysis

Supporting Information A - Individually-optimal Stewardship

Here we derive each manager's individually-optimal stewardship plan and the resulting economic value of a biological resource under individually-optimal stewardship. In so doing, we also formally derive inequality (2) of the main text.

Building on notation presented in the main text: Let γ_t and σ_t denote the actions that the manager will take at time t if the biological resource is in the pristine or evolved state, respectively. The full profile $(\gamma_t, \sigma_t : t = 1, 2, ...)$ is called the manager's 'plan'. Because we are considering a stationary environment (in which annual profits in each state and the likelihood of adverse evolution do not change over time) for the sake of simplicity, it is without loss to focus on plans that are themselves stationary, i.e., $\gamma_t = \gamma$ and $\sigma_t = \sigma$ for all t, and denote them more simply as (γ, σ). Let $\Pi(\gamma)$ and $\underline{\Pi}(\sigma)$ denote annual profit in the pristine and evolved states, respectively, when taking pre-evolution ('stewardship') action γ and post-evolution action σ. Let $\kappa(\gamma)$ denote the annual probability that the resource remains in the pristine state, as a function of the stewardship action γ. Let $V(\gamma, \sigma)$ and $\underline{V}(\gamma, \sigma)$ denote the economic value of the resource in the pristine and evolved states, respectively, when the manager follows plan (γ, σ). Let $V^* = \max_{\gamma,\sigma} V(\gamma, \sigma)$ and $\underline{V}^* = \max_{\gamma,\sigma} \underline{V}(\gamma, \sigma)$ denote the resource's maximal economic value, achieved when the manager follows an individually-optimal plan.

Economic value in the evolved state. Because there is no reversion back to the pristine state (an assumption relaxed below), the resource will generate annual profit $\underline{\Pi}(\sigma)$ once in the evolved state; so, $\underline{V}(\gamma, \sigma) = \underline{\Pi}(\sigma) + \delta\underline{\Pi}(\sigma) + ... = \frac{\underline{\Pi}(\sigma)}{1-\delta}$ and $\underline{V}^* = \frac{\underline{\Pi}(\sigma^*)}{1-\delta}$, where $\sigma^* = \arg\max_\sigma \underline{\Pi}(\sigma)$ is the action that maximizes annual profit once in the evolved state.

Economic value in the pristine state. Suppose that the manager takes stewardship action γ every year so long as the resource remains in the pristine state, then transitions to the optimal post-evolution action σ^* once in the evolved state. In any given year $t \geq 1$, the resource will be in the pristine state, earning annual profit $\Pi(\gamma)$, if it has not experienced adverse evolution in any of the previous $t-1$ years, and otherwise will be in the evolved state, earning annual profit $\underline{\Pi}(\sigma^*)$. Since the annual likelihood of adverse evolution is $1 - \kappa(\gamma)$, the resource will be in the pristine state with probability $\kappa(\gamma)^{t-1}$ and in the evolved state with probability $1 - \kappa(\gamma)^{t-1}$. Accounting for economic discounting of future profits, the present value of the resource in year 1 (what we call its 'economic value') is therefore:

$$V(\gamma, \sigma^*) = \sum_{t\geq 1} \delta^{t-1} \left(\Pi(\gamma)\kappa(\gamma)^{t-1} + \underline{\Pi}(\sigma^*)(1 - \kappa(\gamma)^{t-1})\right) \quad \text{(A-1)}$$

Subtracting $\underline{V}^* = \sum_{t\geq 1} \delta^{t-1}\underline{\Pi}(\sigma^*)$ from both sides, (A-1) can be expressed more simply as

$$V(\gamma, \sigma^*) - \underline{V}^* = \sum_{t\geq 1} (\delta\kappa(\gamma))^{t-1} E(\gamma) = \frac{E(\gamma)}{1 - \delta\kappa(\gamma)} = E(\gamma)L(\gamma), \quad \text{(A-2)}$$

where $E(\gamma) = \Pi(\gamma) - \underline{\Pi}(\sigma^*)$ is the excess annual profit associated with being in the pristine state when taking stewardship action γ and $L(\gamma) = \frac{1}{1-\delta\kappa(\gamma)}$ is the 'effective length' of the pristine phase (defined and used in the main text). We conclude that $V^* = \underline{V}^* + E(\gamma^*)L(\gamma^*)$, where $\gamma^* = \arg\max_\gamma E(\gamma)L(\gamma)$ is the optimal stewardship action.

Note that a manager maximizes the overall economic value of the biological resource by maximizing $E(\gamma)L(\gamma)$, the expected present value of excess profits earned while still in the pristine state. In particular, stewardship action γ is individually beneficial relative to another action γ_0 if and only if $E(\gamma)L(\gamma) > E(\gamma_0)L(\gamma_0)$ or, equivalently, $\frac{L(\gamma)-L(\gamma_0)}{L(\gamma_0)} > \frac{E(\gamma_0)-E(\gamma)}{E(\gamma)}$, verifying our core inequality (2) in the main text.

Extension: allowing for reversion Suppose that, once adverse evolution has occurred, reversion to the pristine state occurs each year with probability $\rho(\sigma)$, depending on the evolved-state action σ. Now, for any given plan (γ, σ), the economic value of the resource in the pristine state $V(\gamma, \sigma)$ and in the evolved state $\underline{V}(\gamma, \sigma)$ are jointly determined by the following system of equations:

$$V(\gamma, \sigma) = \Pi(\gamma) + \delta\left(\kappa(\gamma)V(\gamma, \sigma) + (1-\kappa(\gamma))\underline{V}(\gamma, \sigma)\right) \quad \text{(A-3)}$$

$$\underline{V}(\gamma, \sigma) = \underline{\Pi}(\sigma) + \delta\left(\rho(\sigma)V(\gamma, \sigma) + (1-\rho(\sigma))\underline{V}(\gamma, \sigma)\right) \quad \text{(A-4)}$$

The 'optimal plan' (γ^*, σ^*) is then whatever maximizes $V(\gamma, \sigma)$.

Supporting Information B - An Example Involving Bt Refuge Size

1 Isolated Farmers

We use estimates from the work by Hurley et al. [1] for the US market to parameterize the model. All dollar values are adjusted to 1992 values as in Hurley et al. [1]. The production cost for non-Bt corn is approximately \$185/acre and that for Bt corn is higher, at \$190/acre due costs of producing transgenic plants. Yield for pest-free Bt and non-Bt corn is the same, at approximately 128 bushels/acre. A selling price of \$2.35/bushel therefore gives a total pest-free revenue of \$300.80/acre for both types of crop. Some level of crop loss does occur, however, because of pest infestation. The benefit of a Bt crop is that, by design, it is less prone to loss. Therefore, if we use ϕ_{Bt} and $\phi_{\emptyset}$ to denote the fraction of each crop type that survives pest infestation, then prior to the evolution of Bt resistance we have $\phi_{Bt} > \phi_{\emptyset}$. Once resistance has evolved however we assume $\phi_{Bt} = \phi_{\emptyset}$ and therefore it is no longer worthwhile paying the extra cost of planting Bt crops.

With the above specifications the total annual profit per acre from each type of crop is $\text{Profit}_{Bt} = \$300.80\phi_{Bt} - \$190$ and $\text{Profit}_{\emptyset} = \$300.80\phi_{\emptyset} - \$185$. We assume that, prior to the evolution of resistance, $\text{Profit}_{Bt} > \text{Profit}_{\emptyset}$, meaning that it is best in the short term for a farmer to plant only Bt corn. We next define γ to be the fraction of land set aside for non-Bt crops (i.e., the refuge size) and thus $E(\gamma)$ is the excess profit per year as a result of Bt resistance not yet being present, if the farmer employs a refuge of size γ. This can be calculated by first noting that the annual profit in the pristine state when using a refuge of size γ is $\Pi(\gamma) = (1-\gamma)\text{Profit}_{Bt} + \gamma\text{Profit}_{\emptyset}$. On the other hand, the annual profit in the evolved state, $\underline{\Pi}$, will depend on what the farmer decides to do with the land after resistance evolution has rendered Bt crops no better than non-Bt crops. If the land continues to be used for corn (because doing so is still better than any alternative) then $\underline{\Pi} = \text{Profit}_{\emptyset}$ and thus the excess annual profit is $E(\gamma) = (1-\gamma)\text{Profit}_{Bt} + \gamma\text{Profit}_{\emptyset} - \text{Profit}_{\emptyset}$ or $E(\gamma) = (1-\gamma)(\text{Profit}_{Bt} - \text{Profit}_{\emptyset})$ (see also Hurley et al. [2] equation 4). This is simply the fraction of land planted with Bt corn, multiplied by the excess profit brought about from Bt corn in the absence of resistance. Alternatively, if the land is used for other crops (because other crops are more profitable once resistance has emerged) then $\underline{\Pi}$ will be specific to the crop (and will satisfy $\text{Profit}_{\emptyset} < \underline{\Pi} < \text{Profit}_{Bt}$). The annual excess profit in this case is $E(\gamma) = (1-\gamma)\text{Profit}_{Bt} + \gamma\text{Profit}_{\emptyset} - \underline{\Pi}$. Either expression for $E(\gamma)$ can then be substituted into inequality (2) of the main text to obtain quantitative predictions.

Hurley et al. [1, 2] constructed a detailed biological model that allowed resistance to build up gradually, both within and between years. They used a baseline set of parameter values that resulted in Bt crop failure through resistance evolution in approximately 15 years if no refuge is used. They then showed that, under these conditions, the optimal refuge size is approximately 0.26, meaning that it is economically optimal for a farmer to plant 26% of their crop using non-Bt plants. Doing so reduces the initial annual net income but it prolongs

the time until resistance emerges to beyond 25 years. Numerical sensitivity analyses also revealed that this optimal refuge size is relatively insensitive to the costs and revenues of each type of crop. However, the optimal refuge size increases as the planning horizon increases. When the optimal refuge is used the annualized net income is approximately \$111 per acre.

Comparable results are obtained using inequality (2) of the main text assuming that the farmer continues to plant corn after resistance has emerged (and so $E(\gamma) = (1-\gamma)(\text{Profit}_{Bt} - \text{Profit}_{\emptyset})$). For example, a $\gamma \times 100\%$ refuge will be economically beneficial provided that the effective lifespan of the resistance-free excess profit stream increases by at least $\gamma/(1-\gamma) \times L$ years. Using $\gamma = 0.26$ and the estimate $L = 6$ years for the effective lifespan in the absence of evolution management (see 'Parameter Estimates' below), we obtain $0.26/(1-0.26) \times 6 \approx 2.1$ years. Thus, a 26% refuge will be beneficial provided that it increases the effective lifespan to at least 8.1 years. This corresponds to a Bt-crop failure time through of the evolution of resistance of approximately 22.8 years (see 'Parameter Estimates' below). In other words, inequality (2) of the main text predicts that a 26% refuge will be economically beneficial if it prolongs the time until crop failure from 15 years in the absence of the refuge to at least 23 years in the presence of the refuge. The annual profit under a 26% refuge is predicted to be approximately \$107 per acre which is similar to the \$111 per acre found by Hurley et al. [1, 2]. One can also conduct similar calculations to show that the commonly suggested refuge size of 20% is economically beneficial provided it prolongs the time until crop failure from 15 years in the absence of the refuge to at least 20.3 years in the presence of the refuge.

A second important observation is that, when substituting $E(\gamma) = (1-\gamma)(\text{Profit}_{Bt} - \text{Profit}_{\emptyset})$ into inequality (2) of the main text, we see that whether a refuge is beneficial is independent of the costs and revenues of each crop. Specifically, the right-hand side of inequality (2) of the main text, which is the percentage cost of a refuge of size γ, is simply

$$\frac{\gamma}{1-\gamma} \tag{B-1}$$

This provides an explanation for the findings of Hurley et al. [1, 2] that the optimal refuge size is relatively insensitive to these costs and revenues. On the other hand, if the farmer decides to use the land for other crops once resistance has emerged (because it is more profitable to do so; i.e., $\underline{\Pi} > \text{Profit}_{\emptyset}$) then the right-hand side of inequality (2) of the main text becomes

$$\frac{\gamma}{1-\gamma-\frac{\underline{\Pi}-\text{Profit}_{\emptyset}}{\text{Profit}_{Bt}-\text{Profit}_{\emptyset}}} \tag{B-2}$$

As can be seen, the percentage cost of a refuge of size γ is larger in this case since the consequences of adverse evolution are then smaller. As a result, as $\underline{\Pi}$ increases the use of a refuge becomes increasingly disadvantageous.

Parameter Estimates

Hurley et al. [1] show that, for baseline parameter values, resistance-induced Bt-crop failure in the absence of a refuge occurs after approximately 15 years. Resistance is spreading continually during this entire 15 year period though, causing an increasing amount of crop loss. To approximate this using our approach, which assumes resistance spreads from 0 to 100% at an instant in time, we therefore take the midpoint of 7.5 years as the time to resistance for our model in the absence of a refuge. Thus, an average time to evolution of 7.5 years is obtained in our model by setting $\kappa = 1 - 1/7.5 \approx 0.8666$ for the case of no refuge. Using the 4% economic discounting rate of Hurley et al. [1] gives $\delta = 1/1.04$ and, in the absence of a refuge, the effective lifespan of the excess profit stream is $L = 1/(1 - \delta\kappa) \approx 6$ years.

Similarly, if the effective lifespan of the excess profit stream in the presence of a refuge is 8.1 years, then $8.1 = 1/(1 - \delta\kappa)$ or $\kappa = (1 - 1/8.1)/\delta \approx 0.912$. Thus, the expected time until resistance evolution in our model would be $1/(1 - \kappa) \approx 11.4$ years. Again, taking this as the midpoint for a model such as Hurley et al. [1] this gives a time until resistance-induced Bt-crop failure in the presence of the refuge as 22.8 years.

2 Interacting Farmers

We have focused so far on corn farmers' individual decision whether or not to employ a refuge, taking the parameters of their decision problem as given. In this section, we extend the Bt example to account for the possibility that farmers' decisions can affect one another. In particular, we focus here on the (surprisingly subtle) game-theoretic effects of pest migration [3], especially how (i) migration of *non-resistant* pests can cause the game among farmers to exhibit strategic substitutes and negative externalities while (ii) migration of *resistant* pests can cause the game to exhibit strategic complements and positive externalities.

Suppose there are several farmers growing Bt corn and that none of them has yet had resistance emerge on their farm. For simplicity, suppose that each farmer has a binary choice: to set aside fraction $0 < f < 1$ of their land as a refuge ($\gamma = 1$) or to employ no refuge at all ($\gamma = 0$). Each farmer prefers to employ a refuge whenever the percentage benefit of doing so (i.e., left-hand side of (4) of the main text, denoted B) exceeds the percentage cost (i.e., right-hand side of (4) of the main text, denoted C).

By equation (B-2), the percentage cost C of employing a refuge takes the form

$$C = \frac{f}{1 - f - \frac{\underline{\Pi} - \text{Profit}_\emptyset}{\text{Profit}_{Bt} - \text{Profit}_\emptyset}}, \tag{B-3}$$

where $\text{Profit}_{Bt} = \$300.80\phi_{Bt} - \$190$, $\text{Profit}_\emptyset = \$300.80\phi_\emptyset - \$185$, and ϕ_{Bt} and $\phi_\emptyset$ are the fractions of each crop type (Bt and non-Bt) that survive pest infestation.

Because insect pests can migrate between farms, resistant pests can emerge on a farm in two ways: by evolving *in situ* on the farm (annual probability $1 - \kappa(\gamma)$), or by arriving from another farm where resistant pests have already emerged (annual probability $1 - \eta$). Overall, resistance does not emerge each year with probability $\kappa(\gamma)\eta$. The percentage benefit B of employing a refuge therefore takes the form

$$B = \delta\eta \frac{\kappa(1) - \kappa(0)}{1 - \delta\kappa(1)\eta} \tag{B-4}$$

Note that some of the parameters in equations (B-3, B-4) naturally depend on the choices of other farmers; thus, farmers' decisions constitute a game, the nature of which depends on what sort of impacts farmers' decisions have on one another.

Possibility #1: strategic substitutes and negative externalities. As more farmers in an area employ refuges, the density of non-resistant pests increases, not just on those farms that employ a refuge but on all farms in the area. Farmers who employ a refuge can therefore expect greater losses due to refuge infestation when refuges are in more widespread use; so, $\phi_\emptyset$ (and hence the percentage cost C) is naturally increasing in p; see Figure 3a. Note that if this were the only interpersonal impact of refuge use, the game among farmers would therefore exhibit strategic substitutes (since others' employing refuges increases your own percentage cost of doing so) and negative externalities (since others' employing refuges exposes you to more non-resistant pests, which is never good for profitability).

Possibility #2: strategic complements and positive externalities. As more farmers in an area employ refuges, the emergence of resistant pests is delayed, not just on those farms that employ a refuge but on all farms in the area. Farmers can therefore expect a lower rate of resistant-pest arrival when refuges are in more widespread use; so, η (and hence the percentage benefit B) is naturally increasing in p; see Figure 3b. If this were the only interpersonal impact of refuge use, the game among farmers would therefore exhibit strategic complements (since others' employing refuges increases your own percentage benefit of doing so) and positive externalities (since others' employing refuges exposes you to fewer resistant pests, which is always good for profitability).

We have focused here on two special cases: one in which migration of (only) non-resistant pests causes the game among farmers to exhibit strategic substitutes and negative externalities, and another in which migration of (only) resistant pests causes the game among farmers to exhibit strategic substitutes and negative externalities. Of course, in reality, non-resistant and resistant pests simultaneously migrate. In any realistic model, we should therefore expect the game among farmers to exhibit a complex blend of these two possibilities.

Discussion (additional decisions beyond whether to use a refuge): The game that farmers actually play is far more complex than just whether or not to employ a refuge, as farmers make many other decisions that impact one another. For instance, consider the decision whether to plant only corn or to rotate corn with another lower-revenue crop with different pests. While the farm remains in the pristine state, crop rotation reduces profitability

during years when the other crop is farmed but increases profitability in the years when corn is farmed, since rotation reduces the burden of corn pests [4]. Moreover, because the overall pest burden is lower, other farmers benefit and find it more profitable to farm corn themselves when others rotate their crops. The game among farmers, when deciding whether or not to rotate crops or only plant corn, therefore exhibits strategic substitutes and positive externalities.

Discussion (dynamic considerations): Yet another important source of complexity that we do not address in this paper's analysis relates to game dynamics. For the sake of tractability, we have assumed that each farmer cares only about the *fraction* of others still in the pristine state who invest in stewardship. Of course, farmers naturally care about the *absolute number* of others who remain in the pristine state as well as the fraction of these farmers who are engaging in stewardship. In that richer context, beyond our scope in this paper, the parameters of each farmer's decision problem will naturally vary over time and the game becomes much more challenging to analyze. See Supplementary Information D for further discussion.

Supporting Information C - An Example Involving Fish Farming

This example illustrates how the general results can be applied to find the optimal evolution management strategy when there is a continuum of possible choices. We consider a fish farm where the harvest is structured in cohorts. The farm is stocked with new juveniles at the beginning of a cohort and these individuals grow for some time before they are harvested. During this growth period, some individuals might contract an infectious disease and at harvest a diseased individual is worth only a fraction of a healthy individual.

To control the spread of disease, a farmer can deploy an antimicrobial in the feed. This increases the rate at which infected fish recover from infection. But there is some probability that antimicrobial resistance emerges during a cohort and this depends on the amount of drug used in the feed as well. A farmer must therefore decide how much drug to put in the feed of each cohort. High amounts of drug in a particular cohort result in a high fish recovery rate from infection during that cohort. However, this comes with the risk of driving resistance emergence, thereby reducing the profitability of all future cohorts.

Suppose that a farm is stocked with N new juveniles at the beginning of a cohort at a cost of k per individual. These individuals then grow for a time τ at which point they are harvested. Healthy harvested individuals yield a revenue of b each, whereas infected individuals are worth only a fraction ϵ of this amount (where $0 \leq \epsilon \leq 1$).

Disease transmission during a cohort is modeled using the following pair of differential equations:

$$
\begin{aligned}
\frac{dU}{dt} &= -\beta U I + \gamma I \\
\frac{dI}{dt} &= \beta U I - \gamma I
\end{aligned}
\tag{C-1}
$$

U and I are the numbers of uninfected and infected fish respectively. The constant β is the disease transmission rate and γ is the per capita rate of recovery. We assume that the recovery rate is determined by how much antimicrobial medication the farmer includes in the feed. We also assume that the initial conditions for each cohort are $I(0) = I_0$ and $U(0) = N - I_0$, where $0 < I_0 < N$ and N is the cohort size. Thus, disease-induced death and natural death are both negligible during the cohort, and there are always some infected individuals at the beginning of each cohort (capturing the idea that there is some background source of infection that is always present). Finally, we also assume that $\beta N > \gamma$ for all feasible values of γ, meaning that the disease will spread when rare.

With the above assumptions, the total cohort size will remain constant at N and we can reduce the above pair of equations to a single equation for I:

$$\frac{dI}{dt} = \beta(N - I)I - \gamma I \qquad I(0) = I_0 \tag{C-2}$$

This differential equation can readily be solved, giving the number of uninfected and infected fish at the end of the cohort duration, denoted by $U(\tau;\gamma)$ and $I(\tau,\gamma)$, respectively, with $U(\tau;\gamma) = N - I(\tau;\gamma)$. This notation highlights the fact that the number of uninfected and infected fish at harvest depends on the recovery rate γ.

We suppose that the strategy the farmer must choose during each cohort is the amount of drug to deploy in the feed. Since higher levels of drug deployment result in larger values of γ, we suppose that the recovery rate γ is, itself, the strategy to be chosen. Higher levels of γ come with an higher cost of treatment, however, and we include this using a cost function $c(\gamma)$. Reasonable choices for the function $c(\gamma)$ will satisfy $c(0) = 0$ (the cost of drug is zero if none is used) and $c'(\gamma) > 0$ (the cost increases as the amount drug used increases). It is also reasonable to assume that $c(\gamma)$ is concave up (i.e., $c''(\gamma) > 0$), meaning that *further* increases in the recovery rate are harder to achieve (i.e., more costly) when starting from a higher recovery rate or, in economics jargon, that there are 'increasing marginal costs' associated with achieving speedier recovery.

With these assumptions, the net profit $\Pi(\gamma)$ from a single cohort in the absence of resistance is

$$\begin{aligned}\Pi(\gamma) &= bU(\tau;\gamma) + \epsilon bI(\tau;\gamma) - kN - c(\gamma) \\ &= (\epsilon b - k)N + b(1-\epsilon)U(\tau;\gamma) - c(\gamma)\end{aligned} \tag{C-3}$$

Equation (C-3) has a simple interpretation. First, regardless of infection status, each of the N fish is guaranteed to yield a revenue of ϵb, resulting in a guaranteed profit of $(\epsilon b - k)N$. This is the first term. But each uninfected fish will yield an additional revenue of $b(1-\epsilon)$ over and above the guaranteed baseline revenue of ϵb. This gives the second term, $b(1-\epsilon)U(\tau;\gamma)$. Finally, acquiring this additional revenue by curing fish of infection will incur a treatment cost of $c(\gamma)$.

As in the main text, we simplify the analysis by supposing that the spread of resistance is all or nothing and that, once resistance has spread, the drug is ineffective for all future cohorts. (The analysis here can be augmented to account for the co-evolutionary dynamics of resistant and non-resistant strains within and across cohorts, following Laxminarayan and Brown [5], but the additional complexity resulting from such analysis does not generate any additional insight.) Note that, once resistance has developed, there is no longer any point in using the drug. Consequently, once evolution has occurred, the profit of each subsequent cohort is

$$\underline{\Pi} = (\epsilon b - k)N + b(1-\epsilon)U(\tau;0) \tag{C-4}$$

Lastly, we need to specify how the treatment level (as quantified by γ) affects the likelihood of resistance evolution. If we denote the probability of resistance evolution by $\mu(\gamma)$ (i.e., $\mu(\gamma) = 1 - \kappa(\gamma)$), then it seems reasonable to suppose that $\mu(\gamma)$ is proportional to the total number of treated individuals during a cohort. Therefore, we have $\mu(\gamma) = \nu \int_0^\tau \gamma I(t) dt$ where ν is a (small) positive constant of proportionality. Although this function can be evaluated exactly, the results are more transparent if we suppose that the initial number of infected individuals in each cohort, I_0, is small. In this case, we have

$$\mu(\gamma) = \nu\gamma \frac{1 - e^{-(\gamma - \beta N)}}{\gamma - \beta N} I_0 \tag{C-5}$$

We can see that $\mu(0) = 0$ meaning that the probability of evolution is zero if no drug is used. Furthermore, $\mu(\gamma)$ rises with γ to a peak and then decays such that $\mu(\gamma) \to \nu I_0$ as $\gamma \to \infty$. Therefore, the largest probability of evolution occurs for an intermediate recovery rate [6]. We will denote by γ_c the critical value of γ at which the probability of evolution is the largest. This is the value at which $\kappa(\gamma)$ is the smallest.

Although it is possible to analyze the model under the above assumptions, we make one further simplification. If the epidemiological dynamics are fast relative to the cohort duration, then the number of uninfected and infected individuals reach approximate steady state before the cohort is harvested. In this case, we have $U(\tau; \gamma) \approx \gamma/\beta$.

With this additional simplification, we obtain a very simple expression for the excess annual profit, $E(\gamma) = \Pi(\gamma) - \underline{\Pi}$. We have

$$\begin{aligned} E(\gamma) &= \Pi(\gamma) - \underline{\Pi} \\ &= b(1 - \epsilon)\frac{\gamma}{\beta} - c(\gamma) \end{aligned} \tag{C-6}$$

The first term gives the excess revenue obtained for each fish that is uninfected and the second term is the excess cost incurred to obtain this revenue. Unlike the example of Bt corn, the excess profit function $E(\gamma)$ is a nonlinear function of γ and therefore will involve the actual costs and revenues.

To characterize the optimal management strategy note that, if γ^* is strictly optimal, then from inequality (2) of the main text we must have

$$\frac{L(\gamma^*) - L(\gamma_0)}{L(\gamma_0)} > \frac{E(\gamma_0) - E(\gamma^*)}{E(\gamma^*)} \tag{C-7}$$

for all possible choices of strategy γ_0 that are different from γ^*. There is a continuum of possible strategies and so it can be helpful to work with local conditions by restricting

attention to values of γ_0 that are close to γ^*. In other words, $\gamma_0 \approx \gamma^* + \Delta\gamma$ where $\Delta\gamma$ is small. In this case, assuming both L and E are differentiable, one can approximate the left-hand side of (C-7) (i.e., the percentage benefit of stewardship) as

$$\frac{L'(\gamma^*)}{L(\gamma^*)}\Delta\gamma \tag{C-8}$$

and similarly approximate the right-hand side of (C-7) (i.e., the percentage cost of stewardship) as

$$-\frac{E'(\gamma^*)}{E(\gamma^*)}\Delta\gamma \tag{C-9}$$

If there is an optimal intermediate stewardship strategy $\gamma^* > 0$ then, at this strategy, if a small change in strategy $\Delta\gamma$ is considered, its associated percentage benefit must exactly balance its associated percentage cost; otherwise a one could do better by making a small change. Consequently, if $\gamma^* > 0$, then γ^* must satisfy

$$\frac{L'(\gamma^*)}{L(\gamma^*)} = -\frac{E'(\gamma^*)}{E(\gamma^*)} \tag{C-10}$$

(If $\gamma^* = 0$, then the percentage benefit of a small amount of stewardship must be no larger than the percentage cost, i.e., $\frac{L'(0)}{L(0)} \leq -\frac{E'(0)}{E(0)}$.) Finally, it is interesting to compare the optimal evolution-management strategy characterized by (C-10) with the optimal 'no-stewardship' strategy that simply maximizes $E(\gamma)$. Expressing the left-hand side of (C-10) explicitly we get

$$\frac{\delta\kappa'(\gamma^*)}{1-\delta\kappa(\gamma^*)} = -\frac{E'(\gamma^*)}{E(\gamma^*)} \tag{C-11}$$

Under the assumptions of the current model, $E(\gamma)$ is a unimodal function of γ with a single maximum; so, the optimal evolution-management strategy γ^* will be larger than the no-stewardship optimum γ_0 if $\kappa'(\gamma^*)$ is positive, and it will be smaller than the no-stewardship optimum if $\kappa'(\gamma^*)$ is negative. This can be seen more clearly as follows. Equation (C-11) says that γ^* must occur where $E'(\gamma^*)$ has the opposite sign of $\delta\kappa'(\gamma^*)E(\gamma^*)/(1-\delta\kappa(\gamma^*))$, and so the opposite sign of $\kappa'(\gamma^*)$. For example, it will be negative if $\kappa'(\gamma^*)$ is positive. Now because $E(\gamma)$ is unimodal with a peak at $\gamma = \gamma_0$, having $E'(\gamma^*)$ be negative means that γ^* must be greater than γ_0. An analogous argument holds if $\kappa'(\gamma^*)$ is negative, showing that in this case γ^* must be smaller than γ_0.

To summarize, the evolution-management optimum γ^* will be larger than the no-stewardship optimum γ_0 if $\kappa'(\gamma^*)$ is positive and it will be smaller than the no-stewardship optimum if

$\kappa'(\gamma^*)$ is negative. Which of these occurs will depend on where the minimum of $\kappa(\gamma)$ lies relative to the peak of $E(\gamma)$ (i.e., where γ_c lies relative to γ_0). If $\gamma_0 < \gamma_c$, meaning that the no-stewardship strategy is smaller than that which produces the highest probability of evolution, then we should treat less in order to manage for evolution. In doing so, less excess profit per bout is obtained because more fish are ultimately infected, but this has the advantage of increasing the effective lifespan of the resistance-free profit stream. Conversely, if $\gamma_0 > \gamma_c$ then we should treat more in order to manage for evolution. In doing so, less excess profit per bout is again obtained but now because the cost of achieving this recovery rate is very high. Again, however, this has the advantage of increasing the effective lifespan of the resistance-free profit stream.

Supporting Information D - Manager population dynamics

The annual profitability of a biological resource in the pristine and evolved states, the costs associated with stewardship, and/or the annual likelihood of adverse evolution (hereafter referred to collectively as 'the parameters') all naturally depend not just on (i) what others in the pristine state are currently doing but also (ii) *how many* resources remain in the pristine state and (iii) what others in the evolved state are currently doing. In the main text, we restricted attention to a special case in which managers only decide what actions γ_t to take while in the pristine state, there is no reversion back from the evolved state to the pristine state, and the parameters of each farmer's decision problem only depend on the fraction of others still in the pristine state who engage in stewardship. Under these simplifying assumptions, it is without loss of generality to focus on 'stationary Nash equilibria' in which each manager follows a constant stewardship plan, i.e., $\gamma_t = \gamma$ for all t, even as the environment itself is non-stationary, with more and more resources transitioning to the evolved state.

The stationarity assumed in our main-text model allowed us to present our key findings and insights in the clearest possible way. However, in many contexts, the economics of evolution management is naturally non-stationary. For instance, suppose that corn farmers were to switch immediately to another crop as soon as Bt-resistant pests emerged on their own farms. Under that assumption, the only pests migrating between farms would be non-resistant ones. Moreover, as more and more farms switch away from corn over time, the flow of these non-resistant pests would fall, reducing the cost of employing a refuge (while also reducing the quantity of corn under production and hence increasing the price of corn) and thereby increasing over time farmers' incentive to employ a refuge. In that context, we would expect refuge use to grow more common over time among those farming Bt corn. On the other hand, suppose that corn farmers were to continue to plant (non-Bt) corn after the emergence of Bt-resistant pests on their farms. The burden of Bt-resistant pests would then rise over time, increasing the rate of resistant-pest arrival and thereby reducing farmers' incentive to employ a refuge. In that context, we would expect refuge use to grow less common over time among those farming Bt corn.

To account for such dynamic effects in a model of equal generality as in Supplementary Information A, one would need to allow managers' incentives to change over time as (i) other resources transition back and forth between the pristine and evolved states and (ii) other managers follow a *dynamic plan* $(\gamma_t, \sigma_t : t \geq 1)$ specifying actions in the pristine and evolved states that may change over time. While fascinating and important, a full analysis of such dynamic considerations would require a non-trivial extension of our analytical approach and is beyond the scope of this paper. We therefore leave this for future research.

Supporting Information E - Game-theoretic analysis

In general, to analyze interactions among managers, we can use inequality (2) of the main text but we must allow both L and E to depend on the actions chosen by other managers. To make this clear, we introduce a subscript $\vec{\gamma}$ on the functions E and L to indicate their dependence on the vector of actions taken by all other managers, denoted $\vec{\gamma}$. Thus, in general (2) of the main text is written as

$$\frac{L_{\vec{\gamma}}(\gamma) - L_{\vec{\gamma}}(\gamma_0)}{L_{\vec{\gamma}}(\gamma_0)} > \frac{E_{\vec{\gamma}}(\gamma_0) - E_{\vec{\gamma}}(\gamma)}{E_{\vec{\gamma}}(\gamma)} \tag{E-1}$$

As before, if inequality (E-1) holds, then management action γ is better than management action γ_0. However, whether or not this is true can now depend on others' actions, as specified in the vector $\vec{\gamma}$. In general, the goal of the analysis in this case is to characterize the evolution-management behavior of the entire *population* of managers, accounting for the feedback between managers' choices and their incentives when making those choices. To do so, we view managers' decisions as a 'game' and characterize the Nash equilibria of this game. A list of actions, one for each manager, is called a Nash equilibrium if, when all managers adopt their respective actions, no single manager can do better by unilaterally altering their own action to something else. In some cases, multiple Nash equilibria may exist.

1 Discrete-action case

The simplest analysis assumes that each manager has a set of discrete actions from which they must choose. For example, in the main text we assume that each manager has a binary choice of whether to engage in a fixed level of stewardship (γ) or not (γ_0). We also assumed that both E and L depend on $\vec{\gamma}$ only through a dependence on the fraction of other managers who engage in stewardship (which we denoted by p). We then defined the percentage benefit $B(p) = \frac{L_p(\gamma)-L_p(\gamma_0)}{L_p(\gamma_0)}$ and percentage cost $C(p) = \frac{E_p(\gamma_0)-E_p(\gamma)}{E_p(\gamma)}$ of stewardship.

In the simplest case, if the percentage cost of stewardship is either so small or so large compared to the benefit that managers find it optimal to always or never engage in stewardship regardless of the value of p, then managers are said to have a 'dominant strategy'. More generally, if $C(p) > B(p)$, then it is best for a manager not to engage in stewardship for that value of p, and vice versa if the opposite inequality holds. A *Nash equilibrium* arises when the fraction of managers engaging in stewardship is such that all managers are making an individually-optimal choice. A Nash equilibrium in which all managers take the same action is referred to as a 'pure-strategy equilibrium,' while one in which some managers engage in stewardship and others do not is called a 'mixed-strategy equilibrium' [7]. The set of Nash equilibria can be easily characterized, as follows:

- *No-stewardship equilibrium*: A pure-strategy NE exists in which no managers engage in stewardship (i.e., $p = 0$) if and only if $B(0) \leq C(0)$.
- *All-stewardship equilibrium*: A pure-strategy NE exists in which all managers engage in stewardship (i.e., $p = 1$) if and only if $B(1) \geq C(1)$.
- *Mixed-strategy equilibria*: A mixed-strategy NE exists in which fraction p of managers engage in stewardship if and only if $B(p) = C(p)$.

As long as $B(\cdot)$ and $C(\cdot)$ are continuous functions of p (as we have assumed here), then at least one of the above three cases be true by the Intermediate Value Theorem. Therefore, there is always at least one NE.

Not all Nash equilibria are equally likely to arise in practice. In particular, the population of managers is only ever expected to attain Nash equilibria that satisfy 'convergence stability' [8]. A Nash equilibrium is *convergence stable* if, when the entire population is slightly perturbed from this equilibrium, players have an incentive to change their behavior in a way that brings the population-mix of actions back toward the equilibrium in question. Later, we will discuss convergence stability in more depth in specific cases with strategic substitutes and strategic complements.

Special case: strategic substitutes. The game exhibits strategic substitutes if $B(p) - C(p)$ is strictly decreasing in p. Three possibilities arise. First, if $B(0) \leq C(0)$, then $B(p) < C(p)$ for all $0 < p \leq 1$, managers have a dominant strategy not to engage in stewardship, and the no-stewardship equilibrium is the unique NE. Second, if $B(1) \geq C(1)$, then $B(p) > C(p)$ for all $0 \leq p < 1$, managers have a dominant strategy to engage in stewardship and the all-stewardship equilibrium is the unique NE. Finally, if $B(0) > C(0)$ and $B(1) < C(1)$, then there exists a unique p^* such that $B(p^*) = C(p^*)$ and the mixed-strategy equilibrium in which fraction p^* of managers engage in stewardship is the unique NE.

In each of these three cases, the unique Nash equilibrium is convergence stable, a result that we illustrate here in the case shown in Figure 3a with a mixed-strategy NE. Suppose, for example, that the entire population of managers was perturbed so that the fraction of managers engaging in stewardship was slightly lower than p^*. From Figure 3a, we can see that all managers not engaging in stewardship would then be incentivized to do so because the percentage benefit of stewardship would then be larger than the percentage cost. As a result, the fraction of managers engaging in stewardship would increase, returning the population-mix back toward the mixed-strategy NE.

Special case: strategic complements. The game exhibits strategic complements if $B(p)-C(p)$ is strictly increasing in p. Three possibilities arise. First, if $B(0) \geq C(0)$, then $B(p) > C(p)$ for all $0 < p \leq 1$, managers have a dominant strategy to engage in stewardship, and the all-stewardship equilibrium is the unique NE. Second, if $B(1) \leq C(1)$, then $B(p) < C(p)$ for all $0 \leq p < 1$, managers have a dominant strategy not to engage in stewardship and the no-stewardship equilibrium is the unique NE. Finally, if $B(0) < C(0)$ and $B(1) > C(1)$, then three NE exist: the no-stewardship equilibrium (because $B(0) < C(0)$), the all-stewardship

equilibrium (because $B(1) > C(1)$), and a mixed-strategy equilibrium in which fraction p^* of managers engage in stewardship, where p^* is uniquely determined by the indifference condition $B(p^*) = C(p^*)$.

In the last case, where there are three NE, the two pure-strategy NE are convergence stable but the mixed-strategy equilibrium is convergence unstable. To see why, suppose that the population of managers begins in the mixed-strategy equilibrium but is then perturbed so that the fraction of managers engaging in stewardship is slightly lower than p^*. From Figure 3b, we can see that all managers who are still engaging in stewardship would then be incentivized not to do so, because the percentage benefit of stewardship would then be smaller than the cost. As a result, the fraction of managers engaging in stewardship would decrease, pushing the population-mix further away from the mixed-strategy NE.

2 Continuum-action case

In this section we explain how one can examine cases where there is a continuum of possible strategies, such as choosing the refuge size rather than making a binary decision of whether or not to employ a refuge of a fixed size. For simplicity, we focus on 'symmetric Nash equilibria' in which all managers choose the same evolution-management action $0 \leq \gamma \leq \gamma^{max}$. With a slight abuse of previous notation, let $L_{\hat{\gamma}}(\gamma)$ and $E_{\hat{\gamma}}(\gamma)$ denote, respectively, a biological resource's effective lifespan and excess profit stream in the pristine state when its manager chooses γ and all others choose $\hat{\gamma}$. For a symmetric Nash equilibrium to exist with action γ^*, it must be that

$$\frac{L_{\gamma^*}(\gamma^*) - L_{\gamma^*}(\gamma_0)}{L_{\gamma^*}(\gamma_0)} \geq \frac{E_{\gamma^*}(\gamma_0) - E_{\gamma^*}(\gamma^*)}{E_{\gamma^*}(\gamma^*)} \tag{E-2}$$

for all $\gamma_0 \neq \gamma^*$. This version of inequality (E-1) provides the global condition for characterizing symmetric Nash equilibria. As in Supplementary Information C, a useful first-order necessary condition can be obtained by restricting attention to values of γ_0 that are close to γ^*. Writing $\gamma_0 \approx \gamma^* + \Delta\gamma$ where $\Delta\gamma$ is small, and assuming both L and E are differentiable, we can approximate the left-hand side of (E-2) (i.e., the percentage benefit of stewardship) as $\frac{L'_{\gamma^*}(\gamma^*)}{L_{\gamma^*}(\gamma^*)}\Delta\gamma$ and similarly the right-hand side (i.e., the percentage cost of stewardship) as $-\frac{E'_{\gamma^*}(\gamma^*)}{E_{\gamma^*}(\gamma^*)}\Delta\gamma$. If γ^* arises in a symmetric Nash equilibrium, then these benefits and costs must exactly balance, giving rise to the following first-derivative condition that must be satisfied by any value $\gamma^* \in (0, \gamma^{max})$:

$$\frac{L'_{\gamma^*}(\gamma^*)}{L_{\gamma^*}(\gamma^*)} = -\frac{E'_{\gamma^*}(\gamma^*)}{E_{\gamma^*}(\gamma^*)} \tag{E-3}$$

In other words, the *'marginal cost'* to any single manager of slightly increasing his own action above γ^* must equal the 'marginal benefit' associated with such a change.

Here we characterize all pure-strategy NE when managers are free to choose any stewardship level (also referred to as 'investment level') in the interval $[0, \gamma^{max}]$ and the game exhibits either strategic substitutes or strategic complements. Let $V_{\hat{\gamma}}(\gamma) = L_{\hat{\gamma}}(\gamma)E_{\hat{\gamma}}(\gamma)$ denote the economic value of a biological resource when its manager chooses action γ and all other managers choose action $\hat{\gamma} \in [0, \gamma^{max}]$. For mathematical simplicity, assume that V is continuous and twice differentiable in both γ and $\hat{\gamma}$.

Define $BR(\hat{\gamma}) \equiv \arg\max_{\gamma} V_{\hat{\gamma}}(\gamma)$ to be a manager's best response (i.e., their optimal choice of action) given that all other managers choose action $\hat{\gamma}$. Note that, in general, BR is set-valued since the best response need not be unique. A symmetric pure-strategy NE in this context corresponds to an action, $\gamma^* \in [0, \gamma^{max}]$, such that each manager finds it individually optimal to choose γ^* when all other managers choose γ^*. Or, in mathematical terms, a pure-strategy NE exists with action γ^* if and only if $BR(\gamma^*) = \gamma^*$. Geometrically, γ^* is a value of $\hat{\gamma}$ where the function BR intersects the 45-degree line (also see Figure 4).

To keep the analysis as simple as possible, assume that, no matter what the population-mix of actions (referred to as $\vec{\gamma}$ in the main text), managers have a unique action that maximizes their own individual profit, i.e., managers always have a unique best response. Under this assumption, all managers must take the same action in any Nash equilibrium; so, all NE are symmetric pure-strategy NE.

Let $BR(\hat{\gamma})$ be the manager's unique best response when all others choose $\hat{\gamma}$. $BR : [0, \gamma^{max}] \to [0, \gamma^{max}]$ is referred to as managers' *best-response function.* Assuming that $V_{\hat{\gamma}}(\gamma)$ is continuous, the best-response function will be continuous as well (by a simplified special case of Berge's Maximum Theorem). Furthermore, since the best-response functon is continuous, there exists $\gamma^* \in [0, \gamma^{max}]$ such that $BR(\gamma^*) = \gamma^*$ by the Intermediate Value Theorem; so, at least one NE always exists. The set of NE can be easily characterized:

- *Minimal stewardship*: A NE exists in which all managers invest minimally in stewardship (i.e., $\gamma^* = 0$) if and only if $BR(0) = 0$.
- *Maximal stewardship*: A NE exists in which all managers invest maximally in stewardship (i.e., $\gamma^* = \gamma^{max}$) if and only if $BR(\gamma^{max}) = \gamma^{max}$.
- *Intermediate stewardship*: A NE exists in which all managers invest at intermediate intensity $\gamma^* \in (0, \gamma^{max})$ if and only if $BR(\gamma^*) = \gamma^*$.

As in the discrete-action case, Nash equilibria here may or may not be convergence stable. Provided that $V_{\hat{\gamma}}(\gamma)$ is differentiable, a sufficient condition for convergence stability is $\frac{\mathrm{d}^2V}{\mathrm{d}\gamma^2} + \frac{\mathrm{d}^2V}{\mathrm{d}\gamma\mathrm{d}\hat{\gamma}} < 0$, where this expression is evaluated at $\gamma = \hat{\gamma} = \gamma^*$ [8].

Special case: strategic substitutes. The game exhibits strategic substitutes if $BR(\hat{\gamma})$ is non-increasing in $\hat{\gamma}$. (A sufficient condition for the game to have strategic substitutes is that

$\frac{\mathrm{d}^2 V}{\mathrm{d}\gamma \mathrm{d}\hat{\gamma}} < 0$.) Three possibilities arise. First, if $BR(0) = 0$, then $BR(\hat{\gamma}) = 0$ for all $0 < \hat{\gamma} \leq \gamma^{max}$ and the minimal-stewardship equilibrium is the unique NE. Second, if $BR(\gamma^{max}) = \gamma^{max}$, then $BR(\hat{\gamma}) = \gamma^{max}$ for all $0 \leq \hat{\gamma} < \gamma^{max}$ and the maximal-stewardship equilibrium is the unique NE. Finally, if $BR(0) > 0$ and $BR(\gamma^{max}) < \gamma^{max}$, then there exists a unique γ^* (which is between 0 and $BR(\gamma^{max})$) such that $BR(\gamma^*) = \gamma^*$, and the intermediate-stewardship equilibrium in which managers invest with intensity γ^* is the unique NE. Provided that the function $V_{\hat{\gamma}}(\gamma) = L_{\hat{\gamma}}(\gamma)E_{\overline{\gamma}}(\gamma)$ is differentiable, this value of γ^* must satisfy equation (E-3). Moreover, this intermediate Nash equilbrium is convergence stable.

Special case: strategic complements. The game exhibits strategic complements if $BR(\hat{\gamma})$ is non-decreasing in $\hat{\gamma}$. (A sufficient condition for the game to have strategic complements is that $\frac{\mathrm{d}^2 V}{\mathrm{d}\gamma \mathrm{d}\hat{\gamma}} > 0$.) Three possibilities arise. First, if $BR(0) = \gamma^{max}$, then $BR(\hat{\gamma}) = \gamma^{max}$ for all $0 < \hat{\gamma} \leq \gamma^{max}$ and the maximal-stewardship equilibrium is the unique . Second, if $BR(\gamma^{max}) = 0$, then $BR(\hat{\gamma}) = 0$ for all $0 \leq \hat{\gamma} < \gamma^{max}$ and the minimal-stewardship equilibrium is the unique NE. Finally, if $BR(0) < \gamma^{max}$ and $BR(\gamma^{max}) > 0$, then there exists at least one (and possibly multiple) intensities γ^* such that $BR(\gamma^*) = \gamma^*$. Thus, at least one (and possibly multiple) intermediate-stewardship equilibria exist. Provided that the function $V_{\hat{\gamma}}(\gamma) = L_{\hat{\gamma}}(\gamma)E_{\hat{\gamma}}(\gamma)$ is differentiable, each equilibrium investment level γ^* must satisfy equation (E-3). When there are multiple NE, the equilibrium with the lowest amount of investment ($\gamma^* = \gamma^{low}$) and the equilibrium with the highest amount of investment ($\gamma^* = \gamma^{high}$) are each convergence stable, but other equilibria may be convergence unstable.

Discussion: the impact of subsidies (revisited). For any given $\hat{\gamma}$, subsidizing stewardship increases managers' individually-optimal investment level; so, for each $\hat{\gamma}$, $BR(\hat{\gamma})$ is increasing in the size of the subsidy. When the game has strategic substitutes, $BR(\cdot)$ is a downward-sloping curve that intersects the 45-degree line at exactly one point, and slightly increasing $BR(\cdot)$ results in a slight increase in that crossing point. Thus, when the game exhibits strategic substitutes, small subsidies always result in small increases in equilibrium investment. On the other hand, when the game has strategic complements, $BR(\cdot)$ is an upward-sloping curve that may intersect the 45-degree line at several points. In this context, depending on the precise details, slightly increasing $BR(\cdot)$ can change the set of Nash equilibria, potentially resulting in a large change in manager behavior.

The counterintuitive possibility that a small subsidy can have a large impact on behavior in the case of strategic complements is illustrated in Figure S1. In this example, three symmetric Nash equilibria exist when no subsidy is offered: a 'low-investment equilibrium' with investment intensity $\gamma^* = \gamma^{low}$; a 'high-investment equilibrium' with $\gamma^* = \gamma^{high}$; and a 'medium-investment equilibrium' with $\gamma^* \in \left(\gamma^{low}, \gamma^{high}\right)$. The medium-investment equilibrium can be safely ignored because it is convergence unstable, but the low- and high-investment equilibria are each convergence stable and hence could plausibly arise in practice. (Why is the medium-investment equilibrium convergence unstable? Starting from this equilibrium, any perturbation that slightly increases $\hat{\gamma}$ would incentivize managers to further increase their investment level, pushing managers' behavior further away from this equilibrium and toward the high-investment equilibrium, and vice versa for perturbations that slightly lower $\hat{\gamma}$.)

Assume for a moment that managers are playing the low-investment equilibrium and that a subsidy is introduced. The subsidy causes managers' best-response curve $BR(\cdot)$ to increase, with one of two possible effects. First, whenever the subsidy is less than some threshold $\overline{S}$ (which could be quite small), the $BR(\cdot)$ curve continues to cross the 45-degree line in three places and the set of equilibrium outcomes remain qualitatively unchanged. On the other hand, if the subsidy is larger than $\overline{S}$, the $BR(\cdot)$ curve only crosses the 45-degree line once, at a point higher than the original high-investment equilibrium. In this latter case, introducing the subsidy eliminates the low-investment equilibrium, leaving only the high-investment equilibrium. Managers can therefore be expected to react to such a subsidy by 'adapting upwards' until, eventually, they settle collectively on the high-investment equilibrium, a large overall effect.

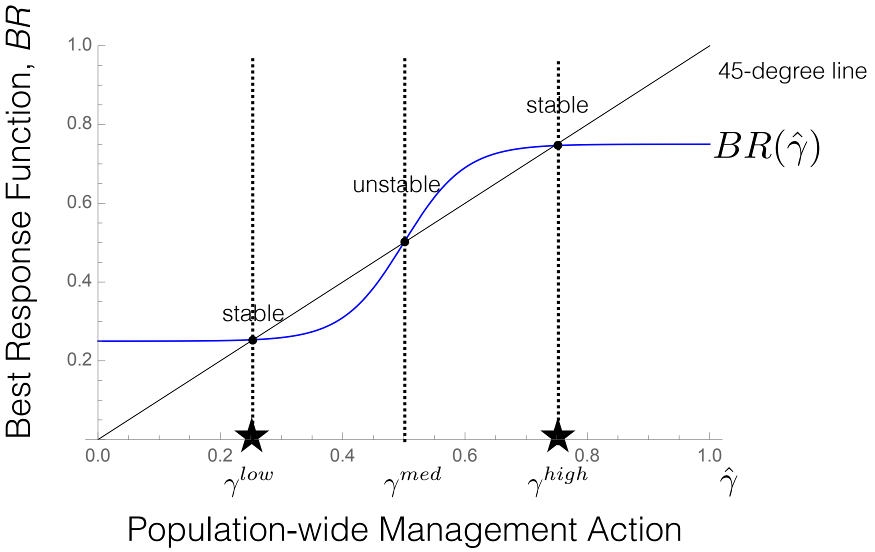

Fig A. An example with a continuum of actions and three symmetric Nash equilibria, with low (γ^{low}), medium (γ^{med}), and high (γ^{high}) levels of investment. Convergence stable values of $\hat{\gamma}$ are indicated by a star. Introducing a subsidy would increase the best-response function $BR(\hat{\gamma})$. When the subsidy is sufficiently large, the low- and medium-investment equilibria disappear, leaving only the high-investment equilibrium.

References

[1] T.M. Hurley, B.A. Babcock, and R.L. Hellmich. Biotechnology and pest resistance: an economic assessment of refuges. *CARD Working papers*, 170:http://lib.dr.iastate.edu/card_workingpapers/170, 1997.

[2] T.M. Hurley, B.A. Babcock, and R.L. Hellmich. Bt corn and insect resistance: an economic assessment of refuges. *J. Agr. Res. Econ.*, 26:176–194, 2001.

[3] J.A. Mutchmor S.A. Coats, J.J. Tollefson. Study of migratory flight in the western corn rootworm (coleoptera: Chrysomelidae). *Environmental Entomology*, 15.3:620–625, 1986.

[4] M. D. Clegg C.A. Francis. Crop rotations in sustainable production systems. *Sustainable agricultural systems*, pages 107–122, 1990.

[5] R. Laxminarayan and R. Brown. Economics of antibiotic resistance: a theory of optimal use. *J. Environ. Econ, and Management*, 42:183–206, 2001.

[6] T. Day and A.F. Read. Does high-dose antimicrobial chemotherapy prevent the evolution of resistance? *PLoS Comp. Biol.*, 12:e1004689, 2016.

[7] A. Dixit, S. Skeath, and D. McAdams. *Games of Strategy, fifth edition.* W.W. Norton, 2020.

[8] I. Eshel. Evolutionary and continuous stability. *Journal of Theoretical Biology*, 103.1:99–111, 1983.
